# Factors That Influence Data Use to Improve Health Service Delivery in Low- and Middle-Income Countries

**DOI:** 10.9745/GHSP-D-19-00388

**Published:** 2020-09-30

**Authors:** Nicole Rendell, Kamalini Lokuge, Alexander Rosewell, Emma Field

**Affiliations:** a Research School of Population Health, Australian National University, Canberra, Australia.; b University of New South Wales, Kensington, Australia.

## Abstract

**Background:**

Health service delivery indicators are designed to reveal how well health services meet a community’s needs. Effective use of the data can enable targeted improvements in health service delivery. We conducted a systematic review to identify the factors that influence the use of health service delivery indicators to improve delivery of primary health care services in low- and middle-income settings.

**Methods:**

We reviewed empirical studies published in 2005 or later that provided evidence on the use of health service delivery data at the primary care level in low- and middle-income countries. We searched Scopus, Medline, the Cochrane Library, and citations of included studies. We also searched the gray literature, using a separate strategy. We extracted information on study design, setting, study population, study objective, key findings, and any identified lessons learned.

**Results:**

Twelve studies met the inclusion criteria. This small number of studies suggests there is insufficient evidence to draw reliable conclusions. However, a content analysis identified the following potentially influential factors, which we classified into 3 categories: governance (leadership, participatory monitoring, regular review of data); production of information (presentation of findings, data quality, qualitative data); and health information system resources (electronic health management information systems, organizational structure, training). Contextual factors and performance-based financing were also each found to have a role; however, discussing these as mediating factors may not be practical in terms of promoting data use.

**Conclusion:**

Scant evidence exists regarding factors that influence the use of health service delivery indicators to improve delivery of primary health care services in low- and middle-income countries. However, the existing evidence highlights some factors that may have a role in improving data use. Further research may benefit from comparing data use factors across different types of program indicators or using our classification as a framework for field experiments.

## Abstract (precis)

We identified factors that may influence the relationship between information generation and improvement of health service delivery: governance (leadership, participatory monitoring, regular review of data); production of information (presentation of findings, data quality, qualitative data); and health information system resources (electronic health management information systems, organizational structure, training).

## Teaser

**Key Findings:**

We identified factors that may influence the relationship between information generation and improvement of health services:

- Governance (leadership, participatory monitoring, regular review of data)
- Production of information (presentation of findings, data quality, qualitative data)
- Health information system resources (electronic health management information systems, organizational structure, training)

**Key Implications:**

- Health system researchers should consider how these factors may apply in the field to build a stronger evidence base for how to effectively translate information drawn from health service delivery indicators into improvements in primary health care service delivery.
- Program managers, district level staff, health facility managers, and health care workers should consider what support they need to use available data to improve decision making at the local level and their role in advocating for improved health service delivery in their communities.

## BACKGROUND

Many countries around the world have developed monitoring and evaluation (M&E) systems to better understand the health of their populations and the effectiveness of their health programs. These systems are intended to capture information about health service delivery to inform how well primary health care services respond to the health needs of a country’s population.^1^^,^^2^ Typically, this is achieved through a series of health service delivery or performance indicators that form part of a broader M&E framework.^1^^–^^3^

The need for M&E has largely been driven by the need for an accountability mechanism in the health system as well as a renewed emphasis on meeting global reporting requirements due to the advent of the Millennium Development Goals and the Sustainable Development Goals.^1^ Numerous organizations and national governments have developed guidance documents to support development and implementation of M&E activities, including those published by the World Health Organization (WHO), the United Nations Develop-ment Programme, and the World Bank.^1^^,^^4^^–^^6^ M&E and its component indicators also play a role in continuous quality improvement, which is grounded by a “data use culture” that promotes the use of evidence to inform decision making.^7^^,^^8^

Health service delivery is the operational end point of the health care system, encompassing the provision of a range of services to promote health in individuals that ultimately lead to positive health outcomes in populations.^9^^,^^10^ Health service delivery indicators are designed to leverage the information obtained through routine data collection to gain greater insights into health services and their capacity to meet the needs of the community. Findings from health service delivery indicators can then be used to drive targeted improvements in health services.^1^^,^^2^ However, the success of this process depends in part on how effectively the indicators are used to generate action where change is needed.

The practice of measuring health system performance against a series of context-specific indicators has long been established. The concept of leveraging data or findings from analyses of a set of indicators to improve health system performance (collectively referred to as “data use” or “data-driven quality improvement”) has drawn some attention in the literature.^11^^–^^15^ The evidence on ways in which data use can be enhanced in practice has mostly focused on vertical programs such as immunization and HIV programs,^15^^–^^18^ rather than having used a horizontal system perspective. However, one broad framework that has received attention in the literature is the Perfor-mance of Routine Information System Manage-ment (PRISM) framework, which incorporates the concept of data use into its assessment tools.^19^ The framework groups determinants of routine health information system (RHIS) performance into 3 categories—technical, behavioral, and organizational factors. The PRISM framework was developed as a theoretical approach, which has since been tested and validated in a range of settings.^19^^–^^23^ To our knowledge, there has been no comprehensive review of practical strategies that can be employed to promote data use at the primary care level of the health system, across all services.

The purpose of our systematic review was to analyze the current literature to identify what factors influence the use of health service delivery indicators to improve delivery of primary health care services in low- and middle-income countries. Specifically, we focused on the factors that serve as barriers or enablers for national and subnational health authorities to use health service delivery indicators in taking action to improve delivery of primary health care services in low- and middle-income countries.

We explored what factors influence the use of health service delivery indicators to improve delivery of primary health care services in low- and middle-income countries.

## METHODS

This systematic review was conducted in line with the Preferred Reporting Items for Systematic Re-views and Meta-Analyses (PRISMA) guidelines.^24^

### Health Service Delivery Indicators

Health service delivery is often described by referencing the type of care (e.g., health promotion, disease prevention, treatment, rehabilitation, palliative care) or the context of the care setting (e.g., ambulatory, primary care, in-patient care).^9^ For the purposes of our review, a health service delivery indicator is a type of health system performance indicator that is produced routinely and focuses on the operational end point of the health system. To answer the research question, we adopted a broad interpretation of “use of health service delivery indicators” by incorporating key terms into our search strategy to reflect the concept of routinely collected health services data such as service delivery indicators, performance indicators, implementation of M&E systems, and data use. At its core, the research question is about the relationship between information and its impact on health care delivery, and broadening the scope of the search strategy captures the wide variation of terminology in the literature.

At its core, our research question is about the relationship between information and its impact on health care delivery.

### Search Strategy and Selection Criteria

We systematically searched 3 databases (Scopus, Medline, and the Cochrane Database of System-atic Reviews) and undertook a Google Advanced Search (first 300 citations, using privacy mode) on March 21, 2020. These databases were selected because they are highly regarded in the field of health systems research. The search terms used were constructed into 3 syntaxes to capture empirical evidence at the primary care level of the health system: (1) [“service delivery indica*” OR “performance indica*”] AND [“health care” OR “health system”] AND [community OR “primary health care” OR “primary care” OR decentrali?ed OR “periph* health cent*”]; (2) [“monitoring and eval*” AND “implement*”] AND [“health care” OR “health system”] AND [community OR “primary health care” OR “primary care” OR decentrali?ed OR “periph* health cent*”]; and (3) “data*” AND [“information culture” OR “information management” OR “decision?making”] AND [“health care” OR “health system”].

A separate strategy was developed to search the gray literature to capture reports published online by national governments or nongovernmental organizations. Such reports were considered likely to contain valuable insights in the use of health service delivery indicators, but they would not have been identified through searching academic databases alone. The strategy for searching the gray literature using Google Advanced Search was adapted from another systematic review by Graham et al.^25^ The search terms for “all these words” were data use, monitoring, evaluation, indicators, and performance. These search terms were combined with the following “exact phrases”: health service delivery, primary health care, and primary care. We also directly searched websites of organizations associated with development assistance including Measure Evaluation; UK Department for International Development; German Office for International Development (GIZ); European Commission–International Cooperation and Development; Japan International Cooperation Agency; and the WHO Alliance for Health Policy and Systems Research. Citations identified through the gray literature search were required to meet the same inclusion and exclusion criteria as those from the databases.

All searches were limited to publication dates between 2005 and present. This timeframe was selected because the first decade of the 2000s marked the beginning of sustained momentum in the development of health information systems and health care quality indicators globally.^26^^–^^28^

We manually reviewed reference lists of the systematic reviews within the field of health systems research (rather than those assessing direct interventions) that were set in low- and middle-income countries, to identify eligible studies for inclusion in the full-text review. The reference lists of all included studies were also searched for further eligible studies.

Studies from both the peer-reviewed and gray literature were eligible if they contained empirical evidence on the use of routinely collected health services data in assessing health care quality at the primary care level. The inclusion criteria also required that studies be available in full text and in English. Studies that were set in high- or upper-middle income countries, according to the *World Bank Country and Lending Groups*,^29^ at the time the study was published were excluded. Studies that did not contain empirical evidence and only canvassed a theoretical discussion of the use of routinely collected health services data were excluded.

Two reviewers independently screened all titles, abstracts, and full-text articles according to the inclusion and exclusion criteria. Differences were resolved by consensus. Where findings of a particular study were reported across 2 papers, only the most comprehensive was selected for full-text review.

### Data Extraction

For each included study, we extracted information on the study design including setting, study population, and study objective. Key findings relating to the use of routinely collected health services data were also extracted, as well as any identified lessons learned that had potential to provide insights into the research question. Only those findings and lessons learned that addressed the research question by describing factors that influenced the use of routinely collected health services data were extracted and synthesized for analysis.

### Quality Assessment

We used the Mixed Methods Appraisal Tool (MMAT) (v-2018, McGill University, Montreal Canada)^30^ to undertake a quality assessment. The MMAT was chosen as the framework to assess quality due to its flexibility in assessing different types of empirical studies. Two reviewers independently appraised each of the included studies. We adopted a similar approach to Burnett et al (2018) and classified studies according to the following^31^:

- High quality, if more than 90% of the relevant criteria were met
- Medium quality, if between 60% and 90% of the relevant criteria were met
- Low quality, if between 30% and less than 60% of the relevant criteria were met
- Very low quality, if less than 30% of the relevant criteria were met

We did not undertake an individual assessment of bias for each of the included studies because of the qualitative design of our review.

### Data Analysis

Themes were derived by undertaking a content analysis of extracted data. Identified themes were considered an enabler if they supported, facilitated, or improved the use of routinely collected health services data to take action to improve service delivery. They were considered a barrier if they restricted, constrained, or prevented this process.

## RESULTS

We identified 7,393 articles through the peer-reviewed literature search and an additional 289 records from other sources. After the removal of duplicates, 7,340 articles remained and were screened based on their title and abstract. Of these articles, 7,321 were excluded. We assessed 19 full-text articles for eligibility, of which 7 articles were excluded because there were no outcomes relating to use of health services data (n=6) or the study design presented only a theoretical discussion (n=1) (Figure). We identified a total of 12 records from database searches and other sources that met the inclusion and exclusion criteria. No un-published or in-process studies were identified. An additional 6 reports meeting the inclusion criteria were identified through the manual gray literature search; however, because all 6 reports were from the same source (Measure Evaluation), they were analyzed separately due to bias concerns.

**FIGURE.**
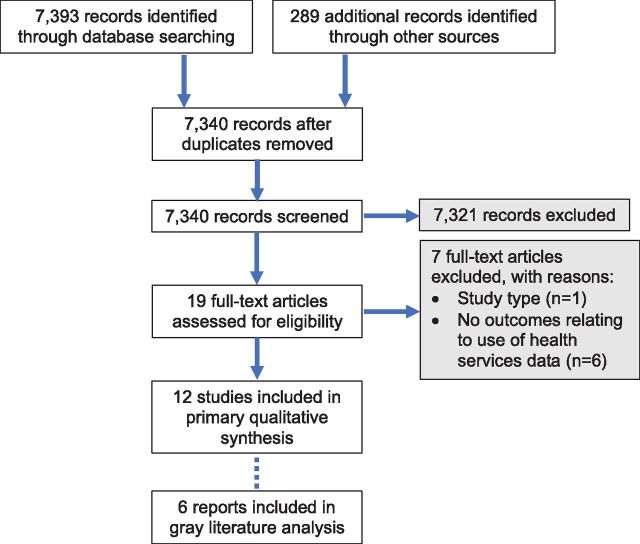
Screening Process Used for Systematic Literature Review of Evidence on Use of Health Service Delivery Data at the Primary Care Level in Low- and Middle-Income Countries

### Study Designs

The majority of the studies that we identified had a cross-sectional design and were conducted in a range of settings. Our sample included a study based in a subnational area of Nigeria^32^; a study on a national level in Afghanistan that analyzed changes in health system performance over a 5-year period^33^; a study based in 3 districts of Cambodia reporting on implementation of an initiative centered around performance-based financing^34^; a study based in a subnational area of India that evaluated a well-established health management information system^35^; and an evaluation based in Côte d’Ivoire assessing change in quality, availability, and use of data following an intervention that aimed to improve RHIS performance.^36^ In addition, there was 1 randomized controlled trial set in Uganda that examined the effectiveness of a community monitoring intervention by comparing communities that received the intervention with communities that did not.^37^ The remaining identified studies were either case studies or qualitative studies that were set in Afghanistan, Cambodia, Kenya, Mozambique, Zambia, Rwanda, and Uganda^38^^–^^43^ (see Supplement for a summary of findings).

A range of recurring themes emerged from the included studies on data use in practice at the primary care level and the associated factors that enhanced the response to findings from health service delivery, performance indicators, or from M&E activities. Each of these is described in Table 1.

**TABLE 1.** Summary of Content Analysis on Factors That Influence Data Use To Improve Health Service Delivery in Low- and Middle-Income Countries

Reference and Location	Peer-Reviewed Literature Analysis									Gray Literature Analysis	
	Leadership	Participatory Monitoring	Presentation of Findings	Data Quality	Qualitative Data	Electronic HMIS	Organizational Structure	Contextual Factors	PBF	Regular Review^a^	Training^a^
**Peer-reviewed literature analysis**											
Björkman and Svensson^37^ – Uganda		X									
Chukwuani et al^32^ – Enugu State, Nigeria					X						
Edward et al^33^ – Afghanistan	X		X								
Edward et al^39^ – Afghanistan		X	X								
Holvoet and Inberg^38^ – Rwanda and Uganda	X			X			X		X	X	
Jacobs et al^40^ – Kirivong Operational Health District, south east Cambodia	X								X		
Kananura et al^41^ – 3 districts in eastern Uganda	X	X			X			X		X	
Khim et al^34^ – Cambodia	X							X			
Krishnan et al^35^ – Ballabgarh, India						X				X	
Nutley et al^42^ – Kenya			X	X							
Nutley et al^36^ – Côte d’Ivoire		X		X		X	X			X	X
Wagenaar et al^43^ – Mozambique, Rwanda, and Zambia		X		X			X				
**Gray literature analysis**											
Afe et al^44^ – Nigeria	X			X		X		X			X
Anasel et al^46^ – Tanzania	X	X		X		X					X
Li et al^45^ – Tanzania	X									X	X
MEASURE Evaluation^47^ – Mali						X					
MEASURE Evaluation^48^ – Kenya	X	X	X							X	X
Millar et al^49^ – Kenya			X			X				X	

Abbreviations: HMIS, Health Management Information System; PBF, performance-based financing.

a Identified as potential factors following gray literature analysis.

A manual search of the gray literature found 6 reports from Measure Evaluation that met the inclusion criteria. Given that these reports were from a single organization, we present the findings from these papers separately in a secondary gray literature analysis.

### Quality Assessment

According to the MMAT quality assessment, most of the studies selected for inclusion in the systematic review were medium quality (n=6), with only 2 being assessed as high quality. The studies identified through the review occupy the lower levels of the evidence hierarchy. Only 1 study had a specified control group and an intervention group as part of the design (a randomized controlled trial), which is the most robust design for comparing strategies that enhance data use.

### Analysis—Peer-Reviewed Literature

#### Leadership

Leadership and the role of active engagement from senior management was highlighted across multiple included studies as a feature associated with strengthened M&E capacity and facilitating uptake at the local level.^33^^,^^34^^,^^38^^,^^40^^,^^41^ The concept of leadership is itself nuanced and difficult to measure and is represented slightly differently in each of the studies. In the study by Holvoet and Inberg,^38^ which compared Rwandan and Ugandan health systems, there was evidence demonstrating the role of leadership in both countries. In Rwanda, strong leadership was identified as a contributing factor in situations in which evidence was used to effectively remedy an issue. Effective governance and strong linkages between processes for planning and M&E were also found to be important factors.^38^ In Uganda, a biannual meeting with ministers and permanent secretaries, in which health sector performance is reviewed and discussed, was shown to improve interest in data quality and use.^38^ Similarly, in another study that was also based in Uganda, Kananura et al^41^ observed that involvement of management at a local level (health district leaders, health facility managers, and subcountry leadership team) in planning and M&E processes strengthened managers’ capacity to use available data to advocate for change. In Cambodia, Khim et al^34^ compared service delivery across 3 districts during the same time period and attributed the success of the highest performing district to strong leadership and management capacity within the district (managers were perceived to objectively undertake performance monitoring). While these 3 studies present insights into leadership and data use, their quality was determined to be low or very low. An earlier study by Jacobs et al,^40^ which was also set in Cambodia, found that improvements in aggregated performance by all health facilities occurred when the district health technical advisory team became more actively involved, highlighting the role of active engagement from senior management. Edward et al^33^ compared service delivery over a 5-year period across most districts in Afghanistan and likewise observed that leadership, specifically the use of “champions,” was an important factor in the successful uptake of a Balanced Scorecard (BSC) as a performance management tool. These studies were of medium and high quality, respectively. Overall, these findings suggest that engaged leadership serves as an enabler for using data on health service delivery, in the form of indicators or M&E findings, to drive action in health service delivery improvement.

Findings suggest that engaged leadership serves as an enabler for using data to improve health service delivery.

#### Participatory Monitoring

Other studies explored the concept of community or participatory monitoring to varying degrees.^36^^,^^37^^,^^39^^,^^41^^,^^43^ A community monitoring intervention formed the cornerstone of Björkman and Svensson’s study^37^ based in Uganda. They found that community monitoring, through dissemination of a report card followed by a series of meetings and joint action planning between the community and health facilities, led to improvements in service utilization and health outcomes. This medium-quality study was the only randomized study with an intervention and control group selected for inclusion in our systematic review. In another medium-quality study, which was based in Côte d’Ivoire, Nutley et al^36^ evaluated the impact of a comprehensive data use intervention. They used dichotomous indicators to assess data use and found it had improved over a 4-year period. The successful intervention incorporated different platforms (e.g., quarterly forums and working groups) to engage a range of stakeholders from data producers to data users.^36^ A high-quality study by Edward et al^39^ also found that participatory monitoring led to positive outcomes, including increased ownership and accountability among those engaged in the process, improved community awareness of rights in accessing health services, and improved service utilization. However, the authors emphasized the important role of facilitation in meetings between community members and health providers to balance their respective demands.^39^ A similar finding was observed by Kananura et al,^41^ who found improved engagement from the community and health services management staff in response to participatory monitoring processes designed to monitor implementation of an antenatal care project. Yet the process was only maintained in the presence of the project team, suggesting challenges exist in ensuring long-term sustainability. Further, the study by Wagenaar et al,^43^ which was conducted at a system level across 3 countries, observed that shared responsibility of data interpretation and collaborative performance review promoted a culture of data use. Both the Kananura and Wagenaar studies were of low quality. All of these findings indicate that participatory (or community) monitoring may serve as an enabler in facilitating the use of health service delivery indicators.

#### Presentation of Findings

More specifically, in terms of presentation of indicator findings, the 2 studies from Afghanistan and 1 study from Kenya provided evidence that data visualization can be used effectively as a performance management tool when combined with leadership and community participation.^33^^,^^39^^,^^42^ The earlier study by Edward et al^33^ reviewed performance trends in 28 of 35 provinces over a 5-year period using a BSC, which served as a means of integrating key performance indicators with benchmarks that align with strategic goals in a range of domains. It was found to be a successful way of assessing and improving health service delivery, although a need for design changes to ensure continued relevance over time was noted.^33^ A later study by Edward et al^39^ examined the feasibility of a variation of the BSC, the Community Scorecard (CSC). A CSC is similar to the BSC, but it seeks community perceptions as part of the analysis and targets the local health care context. Unlike the earlier study by Edward et al,^33^ which was set in 28 provinces, the study by Edward et al^39^ was set in 3 provinces. The authors found that the CSC has potential as a mechanism for enhancing social accountability for quality of care in primary health care facilities, although the CSC development process required strong facilitation by the research team.^39^ Both studies were determined to be high quality. In addition, another type of data visualization, the District Health Profile (DHP) tool, was examined by Nutley et al^42^ in Kenya and found to be effective in facilitating decision making at the district level. The authors suggested that the factors leading to its successful implementation included the tool’s focus on programmatic questions (rather than a long list of indicators) to meet specific information needs of the district and the use of existing technology. Commonly cited barriers among their respondents were a lack of computers and other office equipment such as printers and an underlying lack of value placed on data.^42^ This study was found to be of medium quality. These findings show that the presentation of findings can enhance health service responsiveness to data, although measures need to be taken to ensure ongoing relevance to the given context and availability of appropriate tools. As such, the presentation of findings may serve as a barrier or an enabler depending on the process used to develop the design.

The presentation of findings can enhance health service responsiveness to data, but measures need to be taken to ensure ongoing relevance to the context and availability of appropriate tools.

#### Data Quality

The reliability of routinely collected health services data may affect its use according to 4 of the papers included in our systematic review.^36^^,^^38^^,^^42^^,^^43^ A low-quality study by Holvoet and Inberg^38^ identified poor data quality as a reason for low levels of data use in Uganda. This study described a self-perpetuating cycle in which limited use of data affected the motivation of health facility staff to improve data quality, which then further reinforced the low use. In contrast, Nutley et al^42^ found that implementation of a data visualization tool in Kenya resulted in improvements in data quality even though the primary purpose of the tool was to improve program monitoring to make informed service delivery decisions. This outcome was attributed to the tool helping users identify discrepancies in the data, which could then be corrected.^42^ In a separate study by Nutley et al,^36^ a data use intervention in Côte d’Ivoire that included PRISM assessments and periodic data quality audits was found to be successful in improving data use. Both studies were medium quality. Similarly, Wagenaar et al^43^ advocated for the introduction of data quality assessments as part of any intervention designed to improve data use. They explained that such assessments promote data use in 2 ways: by ensuring confidence in the data, and by showcasing that change is possible through collective effort.^43^ However, this was found to be a low-quality study. While improving data quality is typically perceived as a goal in itself, these findings suggest that data quality is linked to data use. They also suggest that high-quality data may serve as an enabler to facilitate use of health service delivery indicators.

#### Qualitative Data

Qualitative data played a role in supporting quantitative findings in 2 of the included studies.^32^^,^^41^ A study by Chukwuani et al^32^ in Nigeria assessed primary health care operations, using a mix of data collection methods. Qualitative audits of primary health care facilities were found to be valuable in uncovering operational problems because respondents were more pragmatic than in the questionnaire about their needs. For example, results from the staff questionnaire suggested that respondents had knowledge of operational plans and the activity schedule, yet the qualitative audit revealed that their knowledge was limited to immunization activities. Interestingly, the community sample did not have a similar difference, and the authors proposed that a community questionnaire alone could provide sufficient information on its perspective of primary health care operations.^32^ The authors concluded that assessment of primary health care using quantitative data provides valuable information for planning, while qualitative data provide valuable information for understanding effective operations management.^32^ This study was found to be of medium quality. A more recent study by Kananura et al,^41^ which supported these findings, observed that using both qualitative and quantitative data and discussing the results with a diverse group of stakeholders allowed deeper exploration into unanticipated or complex issues. However, this study was found to be of very low quality. These findings suggest that qualitative data represent an enabler for using data to improve health service delivery, when they are part of a broader data collection strategy.

The findings suggest qualitative data represent an enabler for using data to improve health service delivery as part of a broader data collection strategy.

#### Electronic Health Management Information System

The use of an electronic health management information system (HMIS) was captured by 2 of the included studies.^35^^,^^36^ An evaluation of the electronic HMIS was undertaken in Ballabgarh, India, by Krishnan et al.^35^ The authors found that health workers perceived the electronic HMIS as a useful, time-saving means to improve service delivery through development of a monthly work plan based on available data. The authors also found that program managers perceived the electronic HMIS as a better tool for monitoring, supervision, and data management.^35^ These findings are consistent with those of Nutley et al,^36^ who evaluated a data use intervention in Côte d’Ivoire that included implementation of monthly reports from an electronic HMIS at the facility level of the health system.^36^ Both studies were found to be of medium quality. While an electronic HMIS may have the potential to serve as an enabler, in isolation it may be considered as a necessary but insufficient way to enhance the use of health service delivery data to promote health service delivery improvements.

#### Organizational Structure

Few of the included studies identified staffing arrangements as a strategy to improve data use.^36^^,^^38^^,^^43^ The study by Holvoet and Inberg^38^ referred to a specific position at the local level to support M&E activities. These authors identified that appointment and training of data managers in health centers in Rwanda strengthened the local M&E capacity.^38^ However, this study was found to be low quality. The data use intervention found to be successful by Nutley et al^36^ also included M&E-specific positions, and it was determined to be a medium-quality study. The intervention also included additional support for staff such as a leadership program and development of supervision guidelines and data management manuals.^36^ Wagenaar et al^43^ proposed a different perspective by stating that data use interventions should focus on system-wide activities, such as mentoring and supervision and action-planning across all health system actors, rather than on individuals. This study was low quality. These findings point to skilled staff as a possible enabler in supporting the use of health services data to drive change in delivery of health services, although their success may be context specific.

#### Contextual Factors

Two of the included studies noted that contextual factors such as local politics and available re-sources affected the local capacity to respond to findings from indicators or M&E activities, both positively and negatively.^34^^,^^41^ These issues are often deeply entrenched in the local culture. While recognizing that these factors can be influential is important, little can be done in practice to promote or mitigate their impact in the short to medium term.

#### Performance-Based Financing

Performance-based financing (PBF) is a clear application of health service delivery or performance indicators designed to provide improvements in service delivery through financial incentives for health care providers. The implementation of PBF intersects with M&E activities. The study by Jacobs et al^40^ in Cambodia found that the use of performance management in the form of PBF contributed to maintaining a consistent level of health service delivery during a major period of transition. They also found that effective implementation was associated with leadership and was contingent on the M&E activities being undertaken by an independent body.^40^ Furthermore, Holvoet and Inberg^38^ found that PBF reinforced the value of data use at the local level. These findings suggest that PBF could be an enabling factor in the use of data for improvement in health service delivery. However, it would not be implemented as a strategy to improve data use in practice, so it is not useful to consider it a mediating factor in the context of our research question.

### Analysis—Gray Literature

Papers identified through the manual gray literature search were all from the organization Measure Evaluation. These papers formed the basis of a secondary analysis that was conducted separately from the primary analysis of peer-reviewed literature to minimize the risk of bias. If included in the primary analysis, these papers would have constituted a third of the studies and could have influenced the results. In addition, each paper represents an evaluation of a Measure Evaluation project, by Measure Evaluation. While each was subjected to MMAT quality appraisal, this framework does not accommodate questions of independence of the evaluation.

Six reports across 4 countries—Nigeria, Tanzania, Mali, and Kenya—met the inclusion criteria.^44^^–^^49^ According to the MMAT quality assessment, most of the studies selected for inclusion in the gray literature analysis were of high quality (n=3), with only 1 being assessed as low quality.

The major themes identified across the gray literature reports included leadership,^44^^–^^46^^,^^48^ electronic HMIS,^44^^,^^46^^,^^47^^,^^49^ regular reviews of the data,^45^^,^^48^^,^^49^ and training in data use.^44^^–^^46^^,^^48^ This adds weight to the evidence for the role of leadership and electronic HMISs identified in the primary analysis and introduces 2 new potential themes—regular review and training. The gray literature reports also provide evidence to support participatory monitoring,^46^^,^^48^ presentation of findings,^48^^,^^49^ and data quality^44^^,^^46^ as factors in promoting data use. In contrast, the gray literature reports do not provide evidence to support the themes of qualitative data or organizational structure; however, this does not diminish the value of these established themes.

The major themes identified across the gray literature reports included leadership, electronic HMIS, regular reviews of the data, and training in data use.

#### Regular Review

Regular review of program data emerged as a possible factor from the gray literature analysis, and it was subsequently identified as a theme across papers in both analyses. In the analysis of peer-reviewed studies, regular review as a mediating factor in data use was interdependent on other factors, including participatory monitoring or presentation of findings. Consequently, regular review was overshadowed as a standalone theme. Regular data reviews outlined in the gray literature analysis included periodic meetings specifically to understand the data and discuss program performance,^45^^,^^48^^,^^49^ technical working groups,^48^ and stakeholder forums.^48^ These findings from the gray literature analysis suggest regular data review is an underlying factor, and perhaps a necessary or sufficient condition, that may potentially contribute to the use of health service delivery indicators.

#### Training

The role of training and capacity-building activities in data use emerged as an independent theme from the gray literature analysis. Although it did not feature strongly in the primary analysis, it is broadly linked to the theme organizational structure, which was prominent. The evidence suggests that the relationship between data use and training is straightforward—training in data use facilitated staff use of data at the local level,^36^^,^^44^^,^^45^ and conversely, an absence of training was cited as a barrier to data use.^46^ The analysis by Measure Evaluation^48^ in Kenya found that capacity-building activities, which may be considered an extension of training by incorporating ongoing technical assistance and mentoring, resulted in an increased appreciation and ownership of data being used in decision making. This finding suggests that capacity-building activities may have a broad-er reach, facilitating cultural change rather than just an advancement of the technical skillset. Regardless, investment in staff professional development in data use may be an enabler for using health service delivery indicators.

### Classification of Influential Factors on Data Use

The factors identified from our analyses may be grouped into categories to facilitate further discussion and research. We propose 3 groupings: governance, production of information, and health information system resources (Table 2). PBF and contextual factors have been excluded from the classification because they do not represent mediating factors that can be adapted in the short to medium term in practice.

**TABLE 2.** Classification of Influential Factors on Data Use to Improve Health Service Delivery

Governance	Production of Information	Health Information System Resources
Leadership Participatory monitoring Regular review^a^	Presentation of findings Data quality Qualitative data	Electronic HMIS Organizational structure Training (in data use)^a^

a Factors identified through gray literature analysis.

The factors identified from our analyses may be grouped into 3 categories to facilitate further discussion and research.

## DISCUSSION

We identified 12 published studies and 6 reports from a range of low- and middle-income countries that provided empirical evidence on factors that influence the process of using health service delivery indicators to improve delivery of primary health care services. The low number of studies meeting our inclusion criteria suggests that this area of research has received little attention, making it difficult to draw reliable conclusions. Most of the influential factors identified in this setting appeared to serve as enablers. These included the role of leadership in facilitating the use of indicator findings at a local level, participatory (or community) monitoring, presentation of findings, data quality, qualitative data, electronic HMIS, and organizational structure. Regular review of data and training in data use may also have roles to play as independent factors, however, supporting evidence is less clear. The influential factors were grouped into 3 categories for further discussion: governance, production of information, and health information system resources (Table 2). Contextual factors and PBF were each found to have a unique relationship with the use of health service delivery indicators, but they may not be practical to discuss as mediating factors in terms of promoting data use.

### Governance

The studies in our systematic review that discussed the role of leadership referred to engagement of health service managers and senior executives in the planning and evaluation processes at the local level.^33^^,^^34^^,^^38^^,^^40^^,^^41^ The importance of leadership in the context of effective health services management is widely acknowledged,^50^^–^^54^ so it is not surprising that strong leadership may be an enabling factor in promoting use of service delivery indicators. However, the challenge lies in understanding the ways in which leadership capability can be strengthened. Much of the existing literature on leadership in the health sector is focused on individuals. There is scope to undertake more multi-level analyses that consider different team and organizational factors.^54^^–^^56^ One report by the Alliance for Health Policy and Systems Research examined participatory leadership as a strategy for improving health systems.^54^ The report proposed that participatory leadership draws on the collective strength of different actors across the health system that can have a stabilizing impact (reduces vulnerability to actions of individual leaders) or a disruptive impact (challenges the status quo as needed).^54^ This concept aligns with the studies identified in our review that highlight multilevel engagement as a form of strong leadership.

The terms community monitoring and participatory monitoring are used interchangeably and are both forms of social accountability. Social accountability is described by Hamal et al^57^ as “the mechanisms that citizens can use to hold the state and service providers to account for their actions.” Some evidence supports the use of social accountability mechanisms, such as participatory monitoring, to promote quality improvement, particularly at the local level of the health system, although their impact can vary depending on the intervention and/or the context.^57^^–^^61^ Our systematic review identified 4 papers that cite participatory processes that led to positive results. Of note, is the field experiment conducted by Björkman and Svensson,^37^ which investigated community monitoring in Ugandan communities. A follow-up study was conducted 4 years later, and the authors found that the improvements in health care delivery and health outcomes had been sustained in the intervention group that had received the community monitoring support, compared with the control group.^61^ The follow-up study also included a comparison of 2 types of participatory interventions—one with joint action planning alone and the other with joint action planning combined with the dissemination of a report card on staff performance. The findings show that the intervention group that included the report card was more effective.^61^ This outcome suggests that community monitoring activities can serve as a mechanism to promote action in response to service delivery indicators. This approach to accountability may be particularly valuable in settings where government officials are considered ineffective at responding to their own data.

Social accountability such as participatory or community monitoring may support local action in response to findings from health service delivery indicators.

### Production of Information

Our review identified 3 studies that examined 3 different platforms for presenting the findings drawn from monitoring data and service delivery indicators, the BSC, the CSC, and the DHP tool. The literature around BSC and CSC scorecards suggests that CSC is an extension of the BSC, which engages the community and is also perceived as a social accountability mechanism (see previous section on participatory monitoring). Unlike the DHP tool, which was developed as a national solution to support district health data integration in Kenya,^42^ there is evidence to support effective use of both the BSC and CSC in other settings, outside Afghanistan, including high-income countries.^62^^–^^65^ With the advent of electronic HMISs, the opportunities to develop data visualizations such as scorecards and dashboards have grown as has the capacity to measure system logins and data use by health staff. A recent “realist” review of immunization data use undertaken by PATH^16^ found moderate-certainty evidence that decision support tools such as dashboards may improve data use. It also found that such tools are most effective when integrated with established data review and decision-making processes and other forms of feedback such as supportive supervision.^16^

In the studies identified by our review, data quality could be credited with contributing to improvement in the use of health service delivery data, at least in part. While each study proposed an explanation to account for the relationship, it is unclear if the link is due to staff motivation, reassurances in data accuracy, or another factor related to the specific setting of our research question. The realist review by PATH,^16^ which focused on use of immunization data, indicated poor quality may be a barrier to using data, but better data quality does not lead to improved use.^16^ However, there is evidence that suggests the reverse: improved use of data may improve data quality.^16^^,^^66^^,^^67^

In the studies identified by our review, data quality could be credited with contributing to improvement in the use of health service delivery data.

Numerous guidance materials, such as those published by WHO, the World Bank, and the United Nations Development Programme, have advocated for the use of qualitative data as part of a mixed methods design for M&E activities.^1^^,^^4^^,^^6^^,^^68^^,^^69^ The literature also contains many examples of qualitative data being used as part of mixed methods design for M&E activities.^70^^–^^73^ The rationale for using qualitative data is to improve data validity, reliability, and credibility.^68^^,^^69^

### Health Information System Resources

Health information systems form one of WHO’s building blocks and are considered a key part of governance functions.^53^ Electronic HMISs are an efficient tool to serve this function and offer numerous advantages to other systems by ensuring data is quality checked at entry; however, they alone do not ensure data quality and use.^7^^,^^74^^–^^76^ Our review found mixed results in terms of organizational structures to facilitate use of health service delivery data, with 2 studies proposing specific M&E positions and 1 study advocating for investments in system support structures rather than individuals. In terms of improving M&E capability at the local level, the literature has tended to focus on health workers and the importance of feedback mechanisms, supportive supervision, and training in data quality and use, rather than investment in additional trained staff.^7^^,^^14^^,^^16^^,^^66^^,^^75^^,^^77^^–^^79^ This focus is consistent with our gray literature analysis, which identified training as a potential mediating factor in and of itself.

PBF is a supply-side provider payment mechanism that uses financial incentives to motivate individuals and organizations.^80^ Although the concept gained momentum during the early 2000s, particularly in African nations,^81^ its effectiveness in promoting accountability and health system responsiveness versus the impact of unintended consequences is debated.^80^^–^^82^ Evidence is mixed on the effectiveness of PBF in improving health worker performance, service utilization, and health outcomes.^83^^–^^92^ In addition, some evidence highlights the unintended consequences of PBF, such as supplier-induced demand and data manipulation. ^83^^,^^84^^,^^93^ The 2 papers identified in our review highlight the central role of M&E activities as part of implementation of PBF and suggest that PBF reinforces the value of M&E activities. This concept was also highlighted in a review on PBF by Meessen et al.^81^ However, in practice this phenomenon should be perceived as an unintended consequence of PBF because it is impractical to consider the reverse (i.e., PBF as a mediating factor to promote the use of data).

We propose a classification of influential factors identified from our review that may promote the use of health service delivery indicators in our specific setting: governance, production of information, and health information system resources. This classification could be used to structure further work in this space. While our systematic review has identified potential mediating factors at the primary care level of the health system, it is also worth considering the role of system-level approaches to promoting improvements in data use. PRISM assessments (which include data use as part of their framework) have featured in the literature and have been used as an impetus to strengthen RHIS broadly in a range of settings.^19^^–^^23^ Another approach is human-centered design (HCD) although it has featured less prominently.^94^^,^^95^ The HCD approach centers around user needs and applies design thinking principles such as prototyping.^96^ Recent years have seen the introduction of data use partnerships, which aim to build a sustained data use culture. These partnerships are based on a theory of change model that hypothesizes better data and regular data use will create a data use culture, leading to better decisions and improved health outcomes.^97^ The work is funded by the Bill and Melinda Gates Foundation, has been implemented in countries such as Tanzania and Ethiopia using partnerships with local stakeholders, and is in line with the HCD approach.^98^^,^^99^

### Limitations

The main limitation of this review concerns the search strategy. We hypothesized that potentially valuable information is contained in evaluation reports that are published in the gray literature on websites of government agencies or nongovernmental organizations. However, identifying an appropriate methodology that would capture such reports was challenging. During the design phase of this systematic review, different strategies were tested using gray databases and deep web search engines. However, their results could not be repeated for our setting. As such, we decided to opt for a simplified approach using a standard search engine (Google Advanced Search) in privacy mode. Using this approach, no reports were identified that met the inclusion criteria. We also manually searched websites and identified reports that met the inclusion criteria from a single source. To manage the risk of bias we adopted a 2-tier approach to our analyses so the reports that were not published in the peer-reviewed literature were treated as supplementary information, which limited their capacity to distort the results. The search strategy applied limits to the setting (studies in high-income countries were excluded) and primary care level of the health system. While we acknowledge that some observations in high-income countries could likely be applied to low- and middle-income settings, in the interest of responding to the research question, we chose to adopt a narrow scope. This decision may have excluded some studies that should have been included.

Results may also have been limited by the naming conventions of health indicators. For example, indicators reporting on an immunization program may be recorded in the database as immunization indicators or program indicators even though they would also fit the criteria as a performance or service delivery indicator. Further research looking into the application of service delivery indicators may benefit from investigating only program-specific indicators or selecting some key programs and then comparing and contrasting their use across different settings. Alternatively, our classification could be used as a framework to undertake a series of field experiments similar to the methodology of Björkman and Svensson.^37^ This approach supports understanding the weight of each factor, its relationship with other factors, and the effectiveness of system-level approaches.

In addition, we did not undertake an objective assessment of bias for each of the selected studies. The broad range of studies selected for this review meant that the MMAT quality assessment tool was selected to accommodate such differences. Although the MMAT did allow for some level of assessment of bias, comprehensively assessing the risk of bias for each individual study was not possible. The scope of the review may have introduced a publication bias across studies. The research question targets low- and middle-income countries; however, due to limited research capacity in these settings, the literature likely has an underrepresentation of studies from these settings. As such, the experiences of low- and middle-income countries that did not feature in the literature may differ from those published and subsequently captured by the search strategy. The qualitative content analyses may have also introduced an unavoidable risk of both selection and measurement bias.

## CONCLUSION

Scant empirical evidence is available on how health service delivery indicators are used to improve primary health care services in low- and middle-income countries. It is clear there is no single known intervention that could be applied in isolation. However, our systematic review identified some factors that may influence the use of service delivery indicators in practice in low- and middle-income settings: governance (leadership, participatory monitoring, regular review of data); production of information (presentation of findings, data quality, qualitative data); and health information system resources (electronic HMIS, organizational structure, and training in data use). Most of these factors are likely to have an enabling effect. Both contextual factors and PBF were found to have a relationship with the application of health service delivery indicators, but it is not useful to consider these as mediating factors in practice.

Given the narrow scope of the search strategy applied in this review, future research may consider undertaking a broader analysis across different types of program indicators and comparing how these drive change. Alternatively, one could use the classification proposed by this review to test interventions associated with each of the factors in the field to better understand the interrelationships and other possible dominate characteristics that promote translation of data into improved health service delivery at the primary care level of the health system.

## Supplementary Material

19-00388-Rendell-Supplement.pdf

## Notes

**Peer Reviewed**

**First published online:** September 4, 2020
