# Supplementary material for: Factors That Influence Data Use to Improve Health Service Delivery in Low- and Middle-Income Countries
Source: Glob Health Sci Pract. 2020 Sep 30;8(3):566–81. doi: 10.9745/GHSP-D-19-00388 (PMC7541116; doi:10.9745/GHSP-D-19-00388)
Supplement: 19-00388-Rendell-Supplement.pdf [file 19-00388-Rendell-Supplement.pdf]

Supplement to: Rendell N, Lokuge K, Rosewell A, Field E. Factors that influence data use to improve health service delivery in low- and middle-income countries. *Glob Health Sci Pract*. 2020;8(3). https://doi.org/10.9745/GHSP-D-19-00388

Supplement. Summary of Study Findings on Health Service Delivery

Reference	Setting	Study Objective(s)	Findings Related to Application of Health Service Delivery Data[a]	Quality of Evidence (MMAT)
Björkman and Svensson[37]	Uganda	To examine the effectiveness of community monitoring following the introduction of an intervention to support community monitoring	• Community monitoring intervention was associated with increases in utilization of health services and improved health outcomes (reduced child mortality and increased child weight).	Medium<br>(using quantitative randomized controlled trials criteria)
Chukwuani et al[32]	Enugu State, Nigeria	To assess the community profile and needs/expectations, and to evaluate the status of the PHC operations in the state	• Qualitative audits, in addition to questionnaires, in primary health care facilities were valuable in identifying operational issues.<br>• This was not the case for the community sample, and a questionnaire alone may be sufficient for identifying community needs and expectations.	Medium<br>(using mixed methods criteria)
Edward et al[33]	Afghanistan	To illustrate the performance trends in delivering the Basic Package of Health Services during the first 5-year period following elections in 2004 and to reflect on the potential and limitations of the BSC	• The BSC was found to be a valuable tool to assist improvements in the delivery of basic health services 2004–2008.<br>• The design of the scorecard may need to be reconfigured to accommodate changes in the health systems policy and strategy context.<br>• The role of leadership and use of “champions” were considered an important factor in uptake.	High<br>(using quantitative descriptive criteria)
Edward et al[39]	Afghanistan	To assess the impact on service delivery and perceived quality of care following joint engagement of service providers and community members in the design of CSCs	• Facilitating joint provider-community engagement in the development of CSC was found to result in increased ownership and accountability, improved service utilization, and an increased awareness in the community of their rights.<br>• Skilled facilitation is necessary to balance the demands of the community with capacity of the local health system.	High<br>(using qualitative criteria)
Holvoet and Inberg[38]	Rwanda and Uganda	To develop an assessment tool of M&E systems and then apply the assessment	Rwanda:	Low

**Reference**	**Setting**	**Study Objective(s)**	**Findings Related to Application of Health Service Delivery Data[a]**	**Quality of Evidence (MMAT)**
		tool to the health sector M&E systems of Rwanda and Uganda	• Appointment and training of data managers in health centers strengthened local M&E capacity.<br>• PBF in the health sector promoted use of data at the local level.<br>• Instances in which the evidence was used effectively to remedy an issue, contributing factors included strong linkages between planning and M&E, strong leadership, and effective governance.<br>Uganda:<br>• Implementation of a half yearly high-level meeting to discuss health sector performance has improved interest in data quality and use.<br>• Poor data quality was identified as a reason for low data use.	(using qualitative criteria)
Jacobs et al[40]	Kirivong Operational Health District , south east Cambodia	To assess any changes in the level of service delivery, to analyze the underlying reasons for such changes, and to describe the processes used during transition (contracting arrangements from NGOs to a government operated system) to sustain the level of service delivery output	• Use of PBF (performance management tool used to inform performance related pay) was a contributing factor to maintain levels of health service delivery during transitional period.<br>• Improvements in performance by all health facilities was observed when the district health technical advisory team became more actively involved.<br>• Monitoring activities should be conducted by an external or independent agency.	Medium<br>(using quantitative descriptive criteria)
Kananura et al[41]	Three districts in Eastern Uganda	To examine how the participatory M&E approaches were used during the implementation of the MANIFEST project to monitor implementation progress, identify challenges and influence decision making by	• The involvement of health district leaders, health facility managers, and subcountry leadership team in planning and M&E, strengthened their capacity in the use of data for advocacy, planning, and decision making.<br>• Periodically discussing qualitative and quantitative data with a diverse group of stakeholders allowed deeper exploration into unanticipated or complex issues.	Very low<br>(using qualitative criteria)

Reference	Setting	Study Objective(s)	Findings Related to Application of Health Service Delivery Data[a]	Quality of Evidence (MMAT)
		community- and district-level stakeholders	• In the absence of the research team, processes returned to the status quo (prior to the intervention).<br>• Local contextual factors such as a resource availability, political power dynamics and data quality impacted on the actions that could be taken in response to M&E findings.	
Khim et al[34]	Cambodia	To compare the experience in 3 SOA health districts of the process and outcomes of SOA design and implementation and to identify both lessons learnt and areas for policy improvement	• Across 4 primary care indicators there were improvements observed in the 3 districts following SOA introduction. These improvements were not the result of pay for performance contracting alone; there were influences by contextual and national circumstances.<br>• The highest performing district, Memot, experienced stronger improvement in service delivery because of stronger leadership and management capacity in the district.	Low<br>(using mixed methods criteria)
Krishnan et al[35]	Ballabgarh, India	To evaluate the electronic HMIS that has been operational in Ballabgarh for 2 decades	• The health workers perceived that the electronic HMIS saved them time and helped them to improve service delivery, through development of a monthly work plan based on available data.<br>• The program managers perceived electronic HMIS to be a better tool for monitoring, supervision, and data management.	Medium<br>(using mixed methods criteria)
Nutley et al[42]	Kenya	To provide an example of the development and application of a decision-support tool at the district level in Kenya and its effect on data-informed decision making	• The District Health Profile (DHP) tool (a data visualization that responds to 10 health questions and one data quality question with auto-generated graphs by district) was found to improve data-informed decision making at the district level, among those who used it.<br>• The DHP tool was also found to improve data quality which was then reported to enhance data use in decision making.	Medium<br>(using qualitative criteria)
Nutley et al[36]	Côte d'Ivoire	To implement and evaluate an intervention to improve the use of data in decision making in Côte d'Ivoire	• Following implementation of a comprehensive data use intervention, the authors found an increase in use of HIV data in district-level decision making. The intervention activities have been summarized below:	Medium

Supplement to: Rendell N, Lokuge K, Rosewell A, Field E. Factors that influence data use to improve health service delivery in low- and middle-income countries. *Glob Health Sci Pract*. 2020;8(3). https://doi.org/10.9745/GHSP-D-19-00388

Reference	Setting	Study Objective(s)	Findings Related to Application of Health Service Delivery Data[a]	Quality of Evidence (MMAT)
			○ Assess and improve the data use context by conducting PRISM assessments<br>○ Supported quarterly forums, meetings, and working groups to engage data producers and data users<br>○ Periodic data quality audits conducted at national and regional levels with additional resources at the subnational level<br>○ Generation of monthly reports from electronic health records system available at the facility/site level<br>○ Development of a data use plan to identify information needs<br>○ Provision of training (data analysis, interpretation, synthesis, presentation and use) to government health workers, data managers and clinicians<br>○ Strengthen the organizational infrastructure by implementing M&E positions, introduction of a leadership program, and development of resources such as supervision guidelines and data management manuals.	(using quantitative nonrandomized studies criteria)
Wagenaar et al[43]	Mozambique, Rwanda, and Zambia	To describe the similar and divergent approaches to increase data-driven quality of care improvements (QI) and implementation challenges and opportunities encountered in these 3 countries	• The authors found that factors that facilitate data use include:<br>○ A culture in which data quality and interpretation are a shared responsibility and performance review is seen as collaborative and internally driven rather than an external audit.<br>○ Data quality assessments<br>○ Interventions which focus on mentoring and supervision and action planning for all health system actors rather than investment in individuals.	Low<br><br>(using qualitative criteria)
Afe et al[44]	Nigeria	To bridge the knowledge gap concerning the motivators behind using	• The main facilitators to using routine health information in family planning included availability of technology such as computers, data collection and analysis software, reliable	Medium

Reference	Setting	Study Objective(s)	Findings Related to Application of Health Service Delivery Data[a]	Quality of Evidence (MMAT)
		routine health information in FP to improve the use of FP services	internet, and data storage and retrieval systems; staff training in data use; and allocation of funds specifically for data management and use.<br>• Major barriers to using routine family planning data were poor-quality data and the lack of financial resources for supporting quality routine data collection; lack of feedback from higher levels on how data are used; and noninvolvement of policy makers in data collection.	(using mixed methods criteria)
Anasel et al[46]	Tanzania	To understand health providers' capacity to analyze collected family planning data and to document available evidence of health service providers using the collected data in their planning processes	• Barriers to health providers' capacity to analyze and use data were found to include lack of training; poor or no internet connectivity; and lack of office equipment, such as computers.<br>• A missing sense of data ownership led to health providers perceiving they can not use the facility-level data that they are responsible for collecting.<br>• Health providers have no trust in the quality of data at the facility and hesitate to use it during planning.<br><br>A culture of data use was generally not present in the facilities however the authors found that leadership and facilities using the data for budgeting and planning purposes, played a role in creating such a culture.	High<br><br>(using qualitative criteria)
Li et al[45]	Tanzania	To improve data use at the subnational level by building the capacity of subnational regional and council health management teams and district social welfare officers	Key lessons learned to improve data use included:<br><br>• Embedding training on data use with other training programs such as those on new information systems<br><br>• Providing ongoing support for capacity building and peer learning at the local level<br>• Dedicating time to review data to understand program performance<br>• A comprehensive, integrated approach to data use is more effective than a single intervention	High<br><br>(using qualitative criteria)

**Reference**	**Setting**	**Study Objective(s)**	**Findings Related to Application of Health Service Delivery Data**[a]	**Quality of Evidence (MMAT)**
			• Buy-in from leadership is key to institutionalizing data use and providing support for locally driven data use initiatives can further facilitate engagement at the local level.	
MEASURE Evaluation[47]	Mali	To summarize lessons learned and highlight effective health information system strengthening approaches and interventions and their outcomes	• Deploying DHIS 2, an electronic HMIS, that is accessible at multiple levels of the health system, supported improvements in data quality and use.	Medium<br><br>(using qualitative criteria)
MEASURE Evaluation[48]	Kenya	To learn lessons from activities that were implemented in monitoring, evaluating, and communicating the results of the data demand and use interventions associated with the Measure Evaluation PIMA project in Kenya	• Capacity-building activities in data use generated an increased appreciation and ownership for data-informed decision making.<br>• Information products such as visual graphics increased stakeholders' engagement with data.<br>• Collaborative data review meetings improved open communication among the teams about program performance and improved the data availability and accountability.<br>• The development of data-informed strategic documents and guidelines highlighted the value of data for decision making.<br>• Formation of technical working groups and stakeholder forums improved coordination and collaboration on data use activities.	High<br><br>(using qualitative criteria)
Millar et al[49]	Kenya	The study aimed to assess how the MEASURE Evaluation PIMA-supported interventions affected changes in data quality and data use compared with the comparison county.	• Counties that performed well in the analysis developed a costed M&E work plan, with a data use plan, and had regular data review meetings.<br>• In counties with improvement in data use scores, the electronic HMIS has supported the development of health profiles on select indicators and a visual dashboard to support regular review and use of data.<br>• Stakeholders noted concerns about maintaining gains they had made in the absence of project funding.	Low<br><br>(using mixed methods criteria)

Supplement to: Rendell N, Lokuge K, Rosewell A, Field E. Factors that influence data use to improve health service delivery in low- and middle-income countries. *Glob Health Sci Pract*. 2020;8(3). https://doi.org/10.9745/GHSP-D-19-00388

Abbreviations: BSC, balanced scorecard; CSC, community scorecard; HMIS, Health Management Information System; MMAT, Mixed Methods Appraisal Tool; M&E, monitoring and evaluation; PBF, performance-based financing; PHC, primary health care; SOA, special operating agency.

[a] This column refers to any findings or lessons learned that respond the research question by describing factors that influence the use of findings from health services data.
